# Augmenting EEG-global-coherence with auditory and visual noise: Multisensory internal stochastic resonance

**DOI:** 10.1097/MD.0000000000012008

**Published:** 2018-08-21

**Authors:** Ignacio Mendez-Balbuena, Paulina Arrieta, Nayeli Huidobro, Amira Flores, Rafael Lemuz-Lopez, Carlos Trenado, Elias Manjarrez

**Affiliations:** a Institute of Physiology; b Faculty of Psychology; c Faculty of Computational Sciences, Benemérita Universidad Autónoma de Puebla, Puebla, México; d Department of Psychology and Neurosciences, Translational Neuromodulation Unit, Leibniz Research Centre for Working Environment and Human Factors, Technical University Dortmund, Dortmund, Germany.

**Keywords:** auditory noise, coherence resonance, cross-modal stochastic resonance, global coherence, internal stochastic resonance, multisensory stochastic resonance, visual noise

## Abstract

The present investigation documents the electrophysiological occurrence of multisensory internal stochastic resonance (MISR) in the human electroencephalographic (EEG) coherence elicited by auditory and visual noise.

We define MISR of EEG coherence as the phenomenon for which an intermediate level of input noise of a sensory modality enhances EEG coherence in response to another noisy sensory modality. Here, EEG coherence is computed by the global weighted coherence (GWC), modulated by quasi-Brownian noise. Specifically, we examined whether a particular level of auditory noise together with constant visual noise (experimental condition 1) and a specified level of visual noise together with constant auditory noise (experimental condition 2), improves EEG's GWC. We compared GWC between ongoing EEG basal activity (BA), zero noise (ZN), optimal noise (ON), and high noise (HN).

The data disclosed an intermediate level of input noise that enhances the GWC for the majority of the subjects, thus demonstrating for the first time the occurrence of multisensory internal stochastic resonance (SR) in visuoauditory processing within the central nervous system.

## 1 Introduction

Stochastic resonance (SR) refers to a situation in which the addition of noise to the dynamics of a nonlinear system improves its sensitivity.^[1–6]^ SR helps to discriminate weak signals carrying information. SR has been described in a wide variety of physical and biological systems, but its functional significance in the context of internal SR in the human brain remains unexplored. Manjarrez et al^[7]^ showed psychophysical evidence in a yes–no paradigm for the existence of a SR phenomenon in auditory–visual interactions. In particular, they showed that the ability to detect a visual signal by adding auditory noise follows an inverted U-like curve as a function of different noise intensities. However, for a strong visual signal, auditory noise acted in detriment of the visual detection capability. It was also reported that the absolute threshold for the detection of pure tones, in individuals with normal hearing, decreased when a certain amount of noise other than zero is added.^[8]^ Long et al^[9]^ reported in psychophysical experiments that the ability of individuals to detect pure tones near the threshold increases when an optimal noise level is added.

It has been reported that some nonlinear systems in the presence of noise manifest a new type of SR, which is observed in the absence of an external periodic signal. This type of phenomenon has been found in various systems and has been called aperiodic SR,^[10–13]^ autonomous SR^[14]^ or internal SR.^[15–17]^ Because neurons have internally generated noise, it is natural to wonder if these unwanted internal noise sources can be of functional utility. This is an important concept that remains as an open question. To date, experiments designed to study the functional role of internal noise in biological systems have been inconclusive.^[6–18]^

There is a study reporting that when different levels of broadband audible noise are present, observers discriminate between vertical and horizontal luminance.^[19]^ That is, the visual sensitivity profiles of the observers vary according to the different levels of audible noise, which demonstrated a typical function of SR with areas of sensitivity significantly different from basal (without auditory noise conditions). These results show clear evidence that a stochastic synchronization phenomenon is present in the human cortex and that the added noise favors multisensory integration while improving its functionality.^[19]^

The purpose of our study is to demonstrate that electroencephalographic (EEG) activity in humans exhibits the phenomenon of multisensory internal stochastic resonance (MISR) as a consequence of the interaction between the auditory and visual noise of quasi-Brownian type. To our knowledge, there are not studies targeting the interaction between auditory and visual noise in the human brain. Most studies in multisensory physiology employed deterministic signals; however, it is relevant to study the effects of non-deterministic signals (such as noise) as well as their interaction in the multisensory processing. In fact, the vast majority of the visual and auditory sensory inputs that reach our brain are non-deterministic and that is why our study is justified.

In the work of Manjarrez et al,^[17]^ internal SR was shown as a significant increase in the spinocortical coherence when the tactile noise of intermediate intensity was added to the somatosensory system. This phenomenon was termed internal SR since only the addition of noise without an external stimulus gave place to an increase of internal synchrony between spinal and cortical neuronal groups. This leads us to consider whether other sensory signals, besides the tactile ones, can also produce an internal SR phenomenon. To this end, the present study addresses whether interactions between noises of different sensory modalities (visual vs. auditory) generate the phenomenon of internal SR. Specifically, our study contributes to understanding visuoauditory processing via multisensory SR within the central nervous system in the context of electrophysiological signals.

## 2 Materials and methods

### 2.1 Subjects

Nine healthy right-handed subjects (termed S1 to S9 in the Figures; 6 females and 3 males, mean age 23.4 ± 3.7 years) without any history of a neurological disease took part in this study. The female subjects were in different lunar phases.^[20]^ This allowed excluding cyclic ovarian effects on the cortical excitability and oscillatory cortical activity. The tendency to use either the right or the left hand more naturally than the other was tested according to the Oldfield questionnaire.^[21]^ We followed the declaration of Helsinki, and the subjects signed the informed consent. The local ethics committee from the Institute of Physiology (Benemerita Universidad Autonoma de Puebla) approved the experimental protocol.

### 2.2 Experimental paradigm

During the experimental session, the subject sat comfortably in an electrically shielded dimly lit room.

#### 2.2.1 Experimental condition 1 (EC1): visual input noise of constant intensity and modulated audible noise

**Figure 1:**
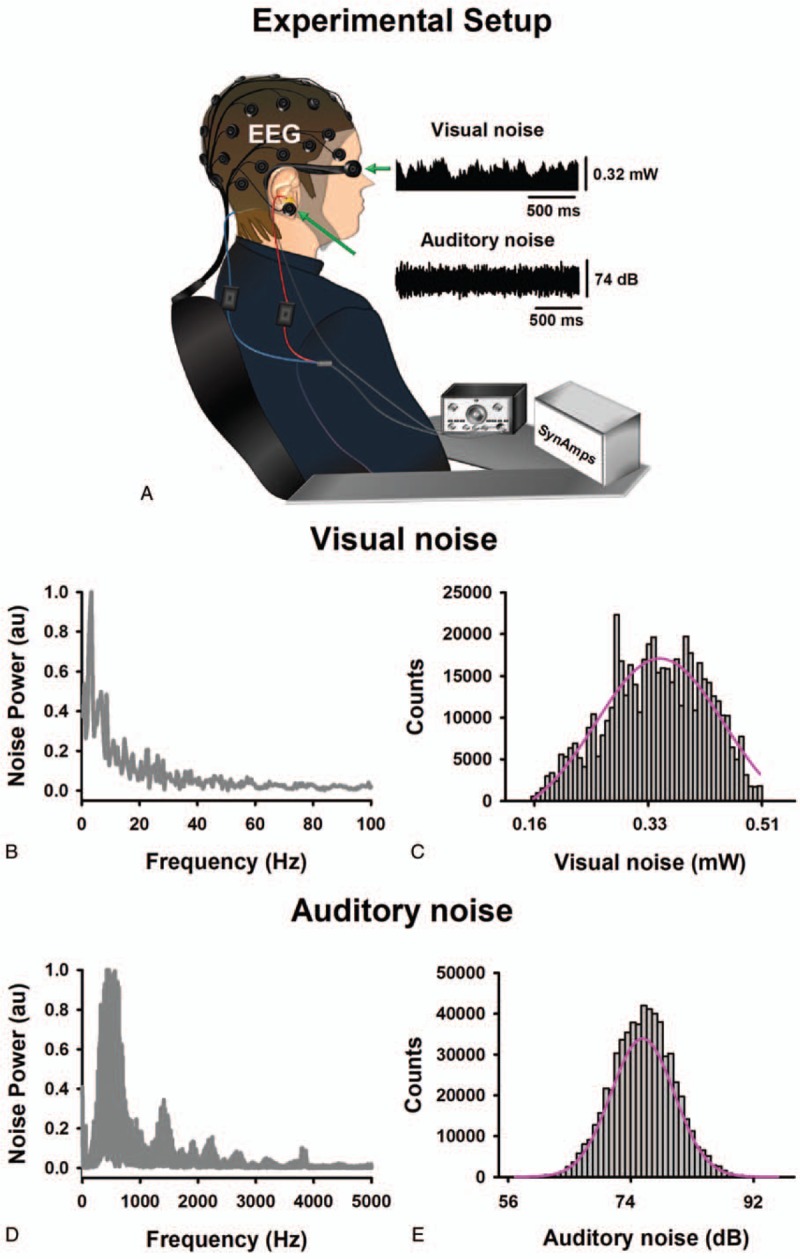
A: Experimental setup. B: Spectral power of visual noise. C: Histogram of visual noise. D: Spectral power of auditory noise. E: Histogram of auditory noise. These spectra were obtained from the noise recordings detected with light (optical power meter PM100D with analog output and sensor type S150C from Thorlabs) and sound (HER-400 Steren) transducers. Here visual and auditory input noise refers to the noise detected by the transducers. Both types of noise followed a Gaussian quasi-Brownian colored noise profile.

The constant visual input noise was calibrated to supply an average value of 0.42 mW, and supplied to the LEDs by a function generator (Tektronix AFG3021C). Five mean intensities of modulated audible noise (56, 65, 74, 85, and 92 dB SPL) stimulated randomly in both ears. The duration of the stimuli was 120 seconds, with 15 seconds inter-stimulus interval. The intensity used was measured using a HER-400 digital decibel meter (Sound Level Meter, Steren) (Fig. 1A).

#### 2.2.2 Experimental condition 2 (EC2): constant auditory input noise plus modulated visual noise

The constant auditory input noise, with an average intensity value of 56 dB, was supplied to hearing devices by using a function generator (Wavetek 132). Five mean intensities of modulated visual noise (0.16, 0.32, 0.40, 0.44, and 0.51 mW) were presented randomly in both eyes. The duration of the stimuli was 120 seconds, with 15 seconds inter-stimulus interval. The intensity used was measured using an optical power meter PM100D with analog output and sensor type S150C from Thorlabs (Fig. 1A).

We defined for each subject a priori, the noise levels which could be considered as ON with the increase of EEG coherence comparison with the ZN. HN was defined when the EEG coherence decreased in comparison with ON. The random signals in both experimental conditions were quasi-Brownian noise type (Fig. 1B–E).

### 2.3 Recordings

The EEG (band pass DC-200 Hz, sampling rate 1000 Hz) was recorded from 30 scalp positions referenced to Cz with the ground at FzA, accordingly to the 10/20 system (SynAmps 2, NeuroScan, El Paso, TX) (Fig. 1A). Electrode impedances were kept under 5 kOhm. The electrooculogram (same bandpass and sampling rate as for EEG) was recorded to exclude trials contaminated with eye movements for further analysis. Auditory and visual input noise signals were recorded in parallel with the electrophysiological data (same bandpass and sampling rate as for EEG). Data were stored and analyzed off-line.

### 2.4 Data analysis

#### 2.4.1 EEG coherence analysis

Only data recorded during the BA, ZN, ON and HN conditions were included for further analysis. Data corresponding to each noise level across trials and subjects were concatenated. Next, the data were further divided into non-overlapping segments. Segments had a duration of 512 ms, therefore allowing a frequency resolution of 1.96 Hz for further spectral analysis. Artifact rejection was visually performed off-line trial by trial. We excluded recordings contaminated with eye movements. We transformed the EEG signal into the reference-free current source density (CSD) distribution that reflects the underlying cortical activity.^[22]^ The CSD algorithm was calculated by using the spherical spline interpolation method^[23]^ implemented in the commercial software “Brain Vision 2.0.1” (München, Germany). For each subject, we obtained 200 segments of artifact-free EEG. The discrete 512 points Fourier transform was computed for each segment by considering the whole frequency band (0–200 Hz).

#### 2.4.2 Calculation of EEG spectral power and EEG coherence

Due that coherence requires the complex values of spectral power (SP), first SP for a given channel (c) was calculated according to the formula: 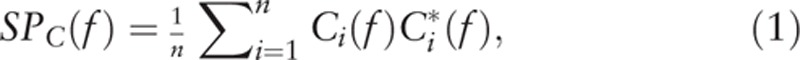


Where *Ci* represents the Fourier transformed channel *C* for a given segment number (*i* = 1,…,*n*) and “∗” denotes the complex conjugate. After this, all 435 possible combinations of coherence values were calculated between pairs of EEG channels to estimate the synchronization between pairs of signals by using the formula: 
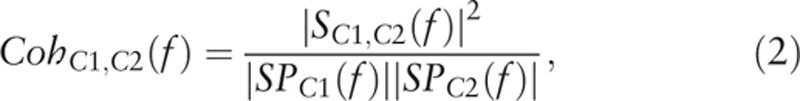


where, 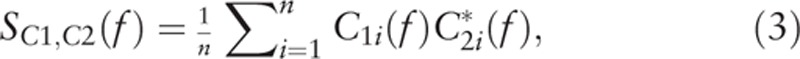


Here *S*_*C*1, *C*2_ (*f*) denotes the cross-spectrum for EEG signal channels *C*1 and *C*2 at a given frequency *f,* whereas *SP*_*C*1_ (*f*) and *SP*_*C*2_ (*f*) denote SP for channels *C*1 and *C*2 at the same frequency. The asterisk represents the complex conjugate. Thus, for frequency *f*, the coherence value, *Coh*_*C*1, *C*2_ (*f*) corresponds to the squared magnitude of a complex correlation coefficient. Note that *Coh*_*C*1, *C*2_ (*f*) is a real number between 0 and 1. We considered that the coherence was significant if the resulting value lies above the confidence level (*CL*).^[24]^ 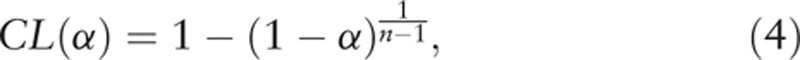


Where *n* represents the number of segments, and the symbol α is the desired level of confidence. We considered coherence to be significantly above the 95% confidence limit (α = 0.95) in the case that *CL* = 0.015 for *n* = 200 segments.

#### 2.4.3 Calculation of Weighted Coherence (WC)

To quantify EEG coherence, we measured the area under the coherence curve and above the significance level. The considered frequency window was 1–45 Hz. For each of the 30 electrodes, we sum up all 29 possible combinations of pairwise coherence to define *WC*_*i*_ for channel *i* as: 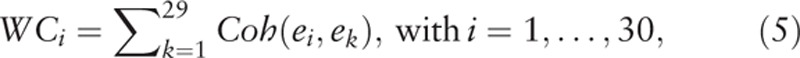


Where, *Coh*(*e_i_*,*e_k_*) denotes the coherence between EEG channels *e*_*i*_ and *e*_*k*_.

To obtain a general measure of the change of EEG synchronization due to a particular level of noise, we define the global weighted coherence (GWC) as: 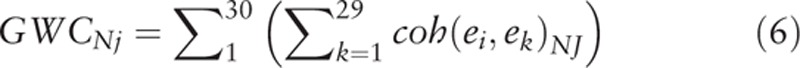


with *i* *=* 1,…,30.

#### 2.4.4 WC maps

To visualize the areas at which *WC*_*i*_, showed an increase in the EEG scalp electrodes, we constructed a topographical head map with each of the 30 values of GWC.

#### 2.4.5 Statistical analysis

To test for any statistical difference in EEG synchronization between particular levels of noise, we computed the GWC for each noise level and condition. As we wanted to study the contrast between ON and BA, ON and ZN as well as ON and HN, statistical comparisons were performed on these conditions. Because our data violated assumptions of normality (Kolmogorov–Smirnov normality test, *P* < .05) and homogeneity of variances (Levene test, *P* < .05), we used non-parametric Friedman analysis of covariance, under the null hypothesis that the dependent variable was the same across factors. Whenever differences were significant, we performed the Wilcox on signed-rank test.

## 3 Results

No subject reported fatigue or anxiety during the experimental sessions.

### 3.1 Effects of multisensory internal SR in EC1: constant visual noise plus modulated auditory noise

**Figure 2:**
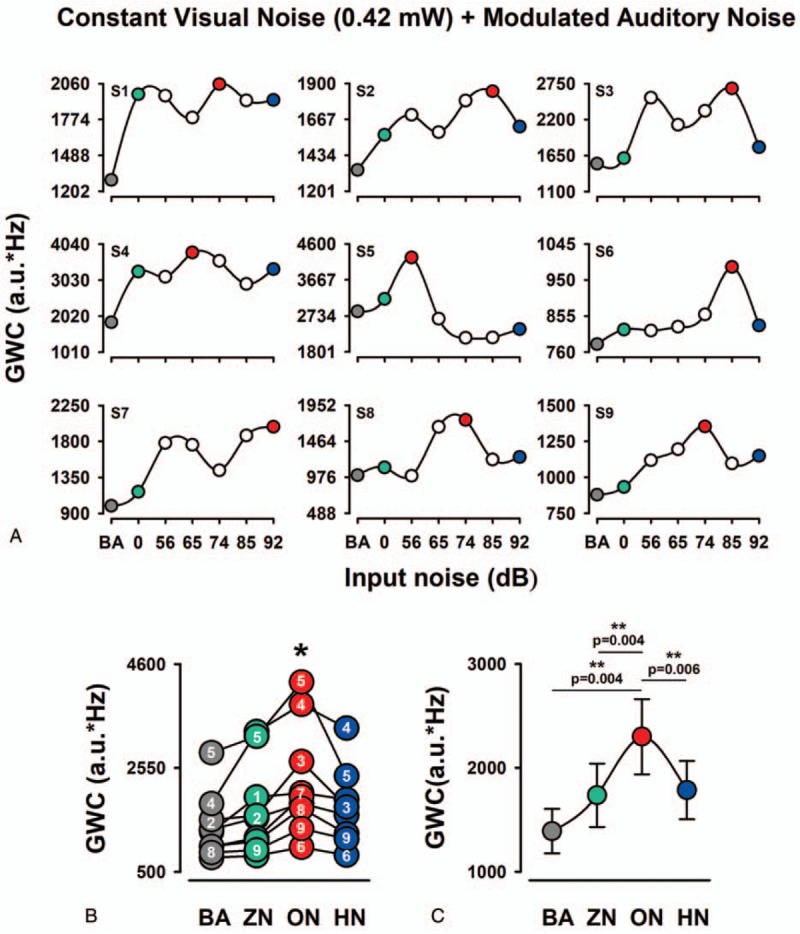
The occurrence of MISR in the GWC of EEG in the EC1. A: Each graph shows the multisensory stochastic resonance effect on the GWC for the BA and 6 levels of visual noise and constant auditory noise. Note that an intermediate level of auditory noise increases the GWC in the majority of subjects. B: Pooled data for the value of GWC from all 9 subjects for BA, ZN, ON, and HN. Within a particular range of input auditory modulated noise, the amplitude distributions of GWC were inverted U-like functions of the input noise. Note the interindividual differences in the GWC values for the noise levels. Numbers inside circles correspond to the subjects. C: Averaged data of GWC. Note the significantly higher amplitude of GWC for ON than for ZN and HN. BA = basal activity, EEG = electroencephalographic, GWC =  global weighted coherence, HN = high noise, ON = optimal noise, ZN = zero noise.

For the majority of the subjects, an intermediate level of auditory input noise enhances the GWC. Moreover, individual differences were found between subjects (Fig. 2A).

We compared the GWC between BA, ZN, ON, HN (Fig. 2B, C). The mean percentage change, between ZN and ON, was 136.85 ± 7.98% (mean ± standard error). Friedman nonparametric test showed a statistically significant change in GWC between the 3 conditions (BA, ZN, ON, and HN): (Ch2 (2) = 18.6, *P* < .001). The Wilcoxon post hoc test revealed statistically significant differences between BA and ON (*P* = .004), ZN and ON (*P* = .004), ON and HN (*P* = .006). In contrast, we did not find statistically significant differences between ZN and HN conditions (Fig. 2C).

**Figure 3:**
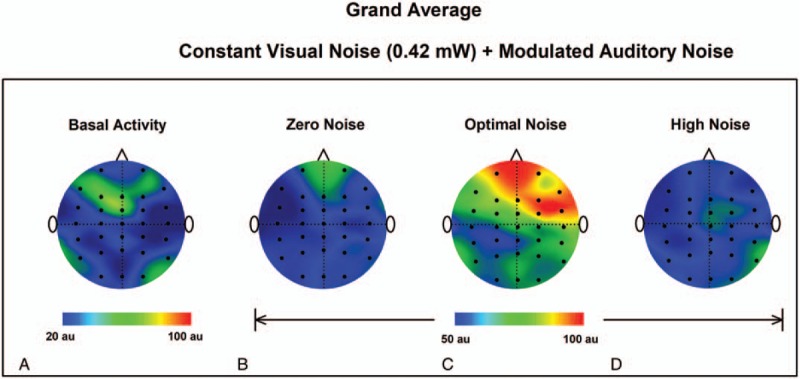
Topographic maps of the GWC grand-average for the experimental condition 1 for the BA, (A) and 3 auditory noise conditions: ZN (B), ON (C), and HN (D). Note that the GWC is higher for ON. BA = basal activity, GWC =  global weighted coherence ,HN = high noise, MISR = multisensory internal stochastic resonance, ON = optimal noise, ZN = zero noise.

We used topographic maps for the qualitative and quantitative representations of the GWC grand average for the BA, ZN, ON, and HN conditions. Note that for the ON condition there are regions of greater synchronization (in the GWC) compared to the BA, ZN, and HN conditions (Fig. 3A–D).

### 3.2 Effects of MISR EC2: constant auditory noise plus modulated visual noise

Analogous to the previous section in this experimental condition, we proceeded to quantify the EEG coherence by the area under coherence curve above the level of significance. The frequency window was 1–45 Hz. We computed all 29 possible combinations of coherence for the 30 electrodes to obtain a general measure of the change in EEG synchronization at a particular level of noise.

**Figure 4:**
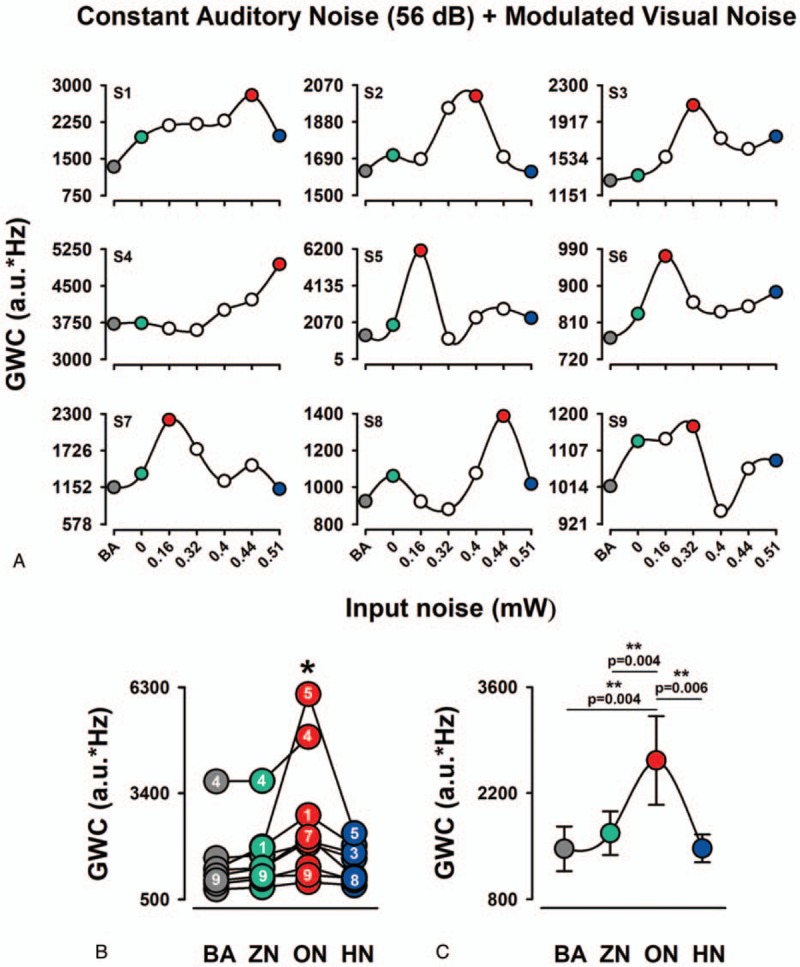
The occurrence of MISR in the GWC of EEG in the EC2. A: Each graph shows the multisensory stochastic resonance effect on the GWC for the BA and 6 levels of auditory noise and constant visual noise. Note that the GWC is characterized by the inverted U shape for the majority of subjects. B: Pooled data for the value of GWC from all 9 subjects for BA, ZN, ON, and HN. Note the interindividual differences in the GWC values for the noise levels. Numbers inside circles correspond to the subjects. C: Averaged data of GWC. Note the significantly higher amplitude of GWC for ON than for BA, ZN, and HN. BA = , EEG =  electroencephalographic, GWC =  global weighted coherence, HN = high noise, MISR = multisensory internal stochastic resonance, ON = optimal noise, ZN = zero noise.

We found that in the majority of the subjects an intermediate level of visual input noise enhances the GWC (Fig. 4A). We compared the GWC between BA, ZN, ON, and HN (Fig. 4B, C). The mean percentage change, between ZN and ON, was 153.33 ± 21.55% (mean ± standard error). Friedman nonparametric test showed a statistically significant change in GWC between the 4 conditions (BA, ZN, ON, and HN): (Ch2 (2) = 20.25, *P* < .001). The Wilcoxon post hoc test revealed statistically significant differences between BA and ON (P = .004), ZN and ON (*P* = .004) and between ON and HN (*P* = .006). In contrast, we did not find statistically significant differences between ZN and HN conditions (Fig. 4C).

**Figure 5:**
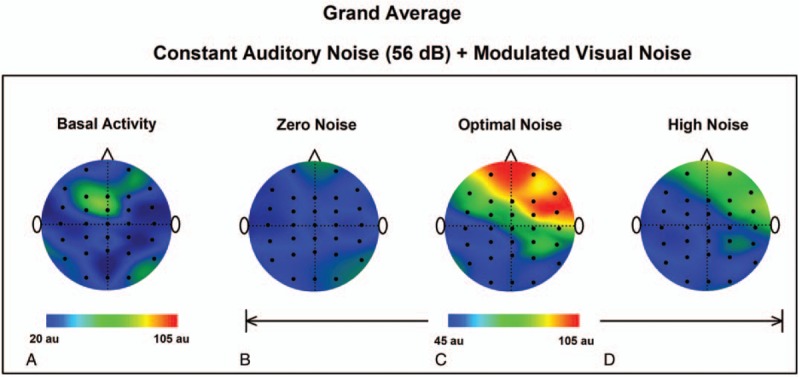
Topographic maps of the GWC grand-average for the experimental condition 2 for the BA, (A) and 3 visual noise conditions: ZN (B), ON (C), and HN (D). Note that the GWC is higher for ON. BA = basal activity, GWC = global weighted coherence,HN = high noise, ON = optimal noise, ZN = zero noise.

In the same way, as in the previous section, topographic maps were constructed for BA, ZN, ON, and HN to obtain a quantitative representation for the GWC grand average. Note that there are regions of higher synchronization in the GWC for the ON condition compared with the BA, ZN, and HN conditions (Fig. 5A–D).

Data Availability: the authors confirm that all data underlying the findings are fully available without restriction upon request. All relevant data are in the paper.

## 4 Discussion

In the present work, we coined a new term, namely “multisensory internal stochastic resonance” (MISR). Our results and approach open new perspectives for the study of multisensory noise interactions in the central nervous system. In what follows, we discuss the main result, which is the increase in cortico-cortical coherence and MISR, due to the interactions of auditory and visual noise.

### 4.1 Constant visual noise and modulated auditory noise

The results obtained in the EC1, demonstrate the phenomenon of multisensory internal SR in the human brain. We observed an increase in GWC for an optimal visual noise level when the modulated auditory noise was presented along with constant visual noise. In the grand average, a more considerable increase is shown in the right frontal area. This aspect was relevant in previous studies demonstrating: coherent oscillations (delta-band), linking frontal, and posterior regions during decision making,^[25]^; theta coherence between frontal and occipital regions during a working memory task^[26]^; and coherence (delta and theta bands) within the frontal region of older adults, related to the memory and executive functions.^[27]^ By such observations, an increase of coherence resulting from MISR is emphasized as a potential approach to modulate coherence in cortical networks with emphasis on frontal regions. In fact, previously we showed that the addition of noise improves motor tasks via an internal SR mechanism associated with a boosting of cortico-muscular coherence.^[28]^

It is worth emphasizing that the mentioned global increase of coherence resulting from MISR may serve the purpose of a marker of brain integrity. For instance, taking the case of a subject that underwent brain damage, we hypothesize that there would probably not be an increase of global coherence in frontal regions or perhaps such increase only occurs in the primary regions of multisensory integration, the cortical occipital-central and temporal-central regions, and the subcortical regions like superior colliculus.^[29,30]^ We hypothesized that the same absence of EEG coherence in frontal regions would occur in anesthetized subjects,^[31]^ as it is not possible for them to compare or discriminate between 2 noisy stimuli applied. Notably, it has also been reported that children with attention deficit hyperactivity disorder (ADHD) exhibited abnormal elevated frontal coherence in comparison to healthy controls,^[32]^ so one would also expect abnormal global coherence resulting from MISR. Further studies will be necessary to characterize the extent to which global coherence changes in subjects with ADHD and the prospect of using such paradigm as a diagnostic tool. It provides advantages: subjects do not need to pay attention or follow instructions that may compromise their results, namely by placing an EEG cap and administering constant visual noise and modulated audible noise, one could find the magnitude of coherence and its brain mapping. In this respect, real-time processing of brain mapping data would be useful.

### 4.2 Constant intensity auditory noise and modulated visual noise

In this experimental condition, we obtained similar results to those described in the previous section. Therefore, we suggest that the physiological processes involved are also similar. We could say that the application of both experimental tests would be very useful in clinical practice, to examine the integrity of brain-associated cognition of noise comparison of different sensory modalities. It is possible that a combination in the addition of noise of other modalities, such as tactile, olfactory or taste could give similar results of an increase in the EEG coherence in frontal areas before the application of a level noise.

Taken together, the findings of both experimental conditions highlight the effect of the interaction of noisy signals not only into primary brain regions and relays of the central nervous system but also in areas of the brain that involve cognitive processes. Note that in the case of anesthetized cats, it was possible to find an internal SR between tactile noise interactions in the spinal cord and the primary somatosensory cortex.^[17]^ It is worth mentioning that for anesthetized cats, an increase in the corticospinal coherence was observed only in the primary cortical processing areas, but not in secondary cortical areas, which might be involved in perceptual processes of tactile sensory information. In this sense, this fact contrasts with what is observed in the present study. The possible explanation for this apparent discrepancy is that the cats were anesthetized and the humans were awake.

### 4.3 Limitations

The first limitation of our study is that the quasi-Brownian visual noise could produce paroxysmal EEG activity in epileptic patients. This is relevant because there is evidence that epileptic EEG paroxysms can interfere with cognitive processes.^[33]^ Therefore, this type of visual noise should be employed with caution in these patients. Furthermore, although we obtained significant results, another significant limitation is the relatively small sample size to separate our statistical analyses by gender or age. Other limitation of our study could be that the developed algorithm for GWC was applied off-line. Therefore, it will be necessary to use other methods with spatiotemporal dynamics to compute the coherence for online applications as in previous studies.^[34]^

### 4.4 Perspectives

It is necessary to see the essence of the findings of this study to describe possible future experiments. We could say that increased EEG coherence in the frontal region, as a result of the application of visual noise and modulated auditory noise, produces in the subject an inalienable propensity for comparison, categorization, or discrimination of the properties of the applied noises. It would be a process of inalienability, very similar to what happens when a figure of a Rubin cup is presented to an awakened subject. In both cases, there is an alternation in perception. Subjects report that they suddenly pay attention to 1 stimulus, or another, in a manner analogous to what happens in the case of Rubin cup. In this case (of Rubin cup), as well as in ours (with the presentation of noises), it is not necessary to instruct the subject to make comparisons. This property of making comparisons emerges spontaneously in an awake and conscious subject. The advantage of our experimental paradigm is that we can calculate the intermediate levels of noise that increase the GWC related to MISR. Such quantification is not possible with the cup of Rubin since it is a constant stimulus. In this context, it is possible that the enhancement of the GWC in MISR can be used as a marker of cognition ability in a subject comparing 2 noise modalities.

## 5 Conclusion

The presentation of visual and auditory noise in awake-subjects produces a significant increase in EEG coherence amplitude in the frontal area of the brain, following the SR phenomenon. This finding is the first evidence of the phenomenon of “multisensory internal-stochastic-resonance.”

## Author contributions

**Conceptualization:** Ignacio Mendez-Balbuena, Nayeli Huidobro, Elias Manjarrez.

**Data curation:** Rafael Lemuz-Lopez, Ignacio Mendez-Balbuena, Elias Manjarrez.

**Formal analysis:** Ignacio Mendez-Balbuena, Paulina Arrieta, Nayeli Huidobro, Amira Flores, Rafael Lemuz-Lopez, Carlos Trenado, Elias Manjarrez.

**Funding acquisition:** Amira Flores, Elias Manjarrez.

**Investigation:** Ignacio Mendez-Balbuena, Paulina Arrieta, Nayeli Huidobro, Amira Flores, Carlos Trenado, Elias Manjarrez.

**Methodology:** Ignacio Mendez-Balbuena, Nayeli Huidobro, Amira Flores, Carlos Trenado, Elias Manjarrez.

**Project administration:** Elias Manjarrez.

**Software:** Nayeli Huidobro, Amira Flores, Rafael Lemuz-Lopez, Carlos Trenado.

**Supervision:** Elias Manjarrez.

**Validation:** Nayeli Huidobro, Amira Flores, Rafael Lemuz-Lopez, Carlos Trenado, Elias Manjarrez.

**Visualization:** Nayeli Huidobro, Amira Flores, Carlos Trenado, Elias Manjarrez.

**Writing – original draft:** Ignacio Mendez-Balbuena, Paulina Arrieta, Nayeli Huidobro, Amira Flores, Carlos Trenado, Elias Manjarrez.

**Writing – review & editing:** Ignacio Mendez-Balbuena, Paulina Arrieta, Nayeli Huidobro, Amira Flores, Rafael Lemuz-Lopez, Carlos Trenado, Elias Manjarrez.

Elias Manjarrez orcid: 0000-0002-3277-0101
